# Optimizations for Energy Efficiency in Software-Defined Wireless Sensor Networks

**DOI:** 10.3390/s20174779

**Published:** 2020-08-24

**Authors:** Sorin Buzura, Bogdan Iancu, Vasile Dadarlat, Adrian Peculea, Emil Cebuc

**Affiliations:** Computer Science Department, Technical University of Cluj-Napoca, 28 Memorandumului Street, 400114 Cluj-Napoca, Romania; sorin.buzura@cs.utcluj.ro (S.B.); vasile.dadarlat@cs.utcluj.ro (V.D.); adrian.peculea@cs.utcluj.ro (A.P.); emil.cebuc@cs.utcluj.ro (E.C.)

**Keywords:** SDWSN, energy efficiency, content aware networking, adaptive data broadcast

## Abstract

Software-defined wireless sensor networking (SDWSN) is an emerging networking architecture which is envisioned to become the main enabler for the internet of things (IoT). In this architecture, the sensors plane is managed by a control plane. With this separation, the network management is facilitated, and performance is improved in dynamic environments. One of the main issues a sensor environment is facing is the limited lifetime of network devices influenced by high levels of energy consumption. The current work proposes a system design which aims to improve the energy efficiency in an SDWSN by combining the concepts of content awareness and adaptive data broadcast. The purpose is to increase the sensors’ lifespan by reducing the number of generated data packets in the resource-constrained sensors plane of the network. The system has a distributed management approach, with content awareness being implemented at the individual programmable sensor level and the adaptive data broadcast being performed in the control plane. Several simulations were run on historical weather and the results show a significant decrease in network traffic. Compared to similar work in this area which focuses on improving energy efficiency with complex algorithms for routing, clustering, or caching, the current proposal employs simple computing procedures on each network device with a high impact on the overall network performance.

## 1. Introduction

The SDWSN paradigm [1] represents the enhancement of the wireless sensor network (WSN) network type with the software-defined networking (SDN) architecture to allow easier network management [2]. As sensor networks are employed for a large variety of applications and their numbers are increasing [3], there is a need that such a network delivers good quality of service (QoS) for the data flows and quality of experience (QoE) for the end user applications [4]. For good QoS and QoE, it is important that the network has a certain degree of autonomy and that it can make decisions on its own with limited human input [5]. Moreover, due to the increased scalability attribute of the SDWSN [6,7], the system must be implemented for the efficiency of energy and data transmission [8]. With their increased capacities, SDWSNs are rapidly becoming an enabler technology for IoT [9,10].

**Figure 1:**
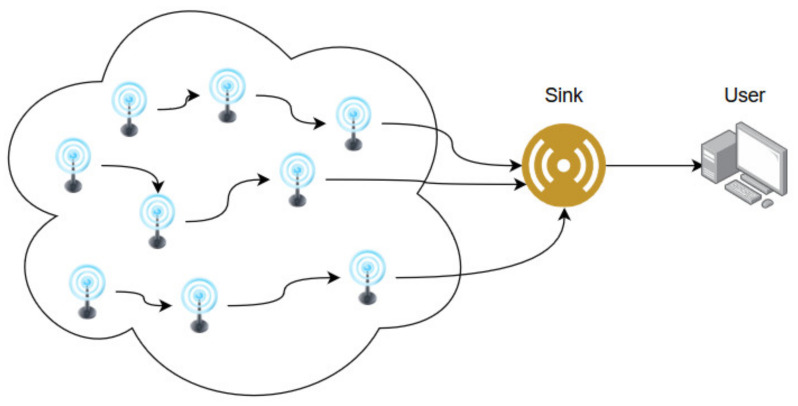
Traditional WSN architecture.

In the traditional SDN architectures, the responsibilities are separated between different planes (or layers), more precisely, data plane, control plane and the applications plane. The data plane consists of physical devices that transfer data by applying the forwarding rules defined by the control plane. The control plane manages the data plane by defining the flow tables and maintaining full knowledge of the entire network topology. In addition, the control plane offers services to the upper applications plane by abstracting the information retrieved from the devices in the data plane. A WSN (Figure 1) is a network consisting of multiple communication nodes which are equipped with one or more sensors and a power supply (usually a battery). The WSN usually serves the function to gather data collected by the sensors and deliver it to a component which analyses or processes the data via a sink node. The SDWSN paradigm integrates a WSN in the data plane of the SDN thus scaling and enhancing the functions of the data plane [1]. Functions that are of interest in the current work are energy efficiency, content awareness, caching, and adaptive data broadcast.

Energy efficiency in WSNs is a very approached topic in the research community [11,12,13]. The current objective is to increase the sensor lifespan by reducing the number of generated data packets in the resource-constrained WSN layer of the system. This work proposes a system where the traffic generation from the WSN data plane is moved to the upper SDN control plane. This is achieved by implementing the following three main ideas. First, the control component is configured with a simple learning and decision-making algorithm to determine if any sensors can be optimized for data transmission. Secondly, the control component caches the last received value from each sensor in order to prepare the data for broadcasting upstream towards the applications plane. The process of broadcasting data in this manner is also encountered in the literature as air caching [14] or air storage [15]. The main purpose of adaptive data broadcast is to deliver the information to the clients before it is being requested. In the current context, the main function of the data broadcast is to improve the energy efficiency, but this can also have a positive impact on the QoS and QoE of the end-users. And thirdly, each sensor is enhanced with content awareness capabilities.

The work becomes more complex as the control component is given more authority to correctly determine which sensors can be optimized to avoid the data transmission. This is achieved by enabling a basic learning and decision-making component in the controller based on data frequency emission for each sensor. The current approach considers time intervals between received packets as processing data for the learning component.

The current research brings a novelty through the fact that data transmission (from the data plane) is replaced by data transmission in the control plane. This is achieved through network programmability employing the concept of adaptive data broadcast of cached data. The cached data consists of the last known value received from each sensor in the control component. This process also enhances the network autonomy as many functions are delegated to the devices which already perform operations in the network. Some limitations exist for the presented findings as the results were obtained through simulations of sensor data and not in a network with real sensors. Nevertheless, these simulations were generated using wireless transmission between real network devices, therefore validating the results and the behavior of data passing sensor data through the network.

The main original contributions of the paper are in the area of energy efficiency in SDWSNs. This is achieved by reducing the number of generated data packets using a novel system architecture with two main concepts:

- content awareness, implemented at the individual programmable sensor level in the resource-constrained sensors plane of the network
- adaptive data broadcast of cached data, using a basic learning component which learns the behavior for each sensor; the technique is used to replace data transmission from the data plane with data transmission in the control plane

The proposed approach is efficient in increasing the overall network lifetime even in environments with high packet loss ratio and also improves the QoS of SDWSNs and the QoE for end-user applications.

The paper is structured as follows: Section 2 provides background knowledge together with related work which influenced this research. Section 3 presents the system design and the proposed algorithms. Section 4 presents details about the testing environment. Section 5 discusses the obtained results and applicability of the system in different environments. Section 6 concludes the paper and proposes some ideas for future research.

## 2. Related Work

With the evolution of wireless sensor networks over time, the mechanisms which ensure effective data transmission and longer sensor lifespan have been required to evolve as well to match the end-user applications’ expectations [16,17]. Improvements are currently being developed in enhancing the programmability of such networks. Consequently, the created environments become more manageable and autonomous leading to the usage of different network topologies and technologies [3,4,5,18]. The topics covered in this chapter are energy awareness in WSNs, content-aware networking, WSN data routing mechanisms, security of data transfer in WSNs, data caching, and adaptive data broadcast. The relationship of each topic is also detailed in the context of the SDWSN environment and related studies are presented, highlighting the contributions brought by the current work. 

**Figure 2:**
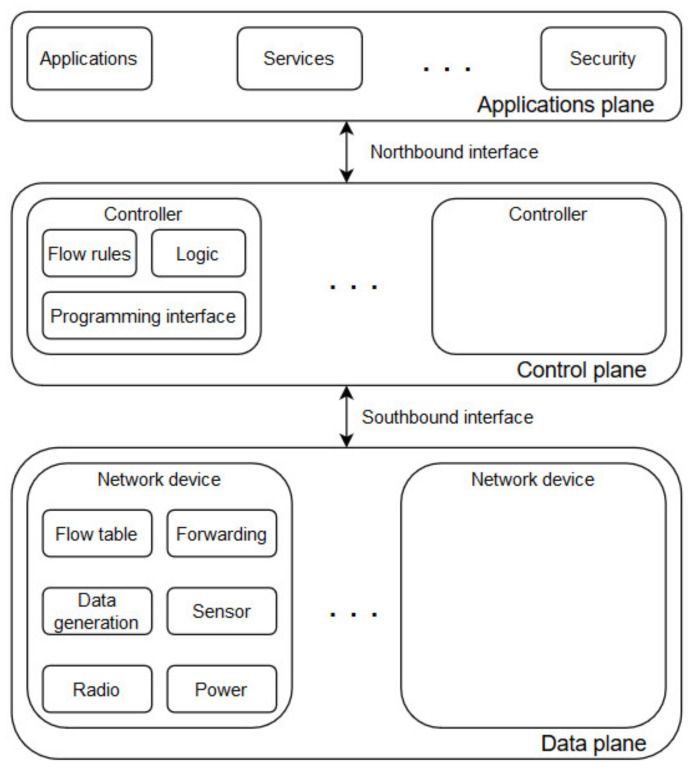
Traditional SDWSN architecture.

Figure 2 presents the general SDWSN concept and it can be regarded as an extension over the SDN architecture. The control plane remains responsible to programmatically manage the network and to maintain full knowledge of the entire network topology. However, the data plane complexity is increased with multiple technologies and more dynamic data flows. The SDWSN architecture does not impose the use of any specific communication protocols, but it still allows integrating commonly used SDN protocols, such as OpenFlow [19], for defining packet forwarding rules.

The layered terminology also mentioned northbound and southbound interfaces. A northbound interface allows a network component to communicate with another component belonging to a higher abstraction level, whereas a southbound interface allows a network component to communicate with a component from a lower level. The key ideas of this separation are to make network management easier and to reduce the network overhead. The network overhead is reduced by moving some computations in the control plane whereas the data plane remains responsible only for data generation and data forwarding. An SDN enabled WSN will have a more complex data plane due to the presence of many nodes and, possibly, due to the heterogeneity of the nodes [2]. The control plane remains responsible for the complete network topology overview and management; however, some logic or algorithms will have to be moved (not necessarily entirely) in the data plane, as is the case of the current work. In the proposed approach, the concept of content awareness is delegated to the data plane at each individual sensor node, but the logic controlling the content handling remains implemented in the control plane.

One of the most important problems that WSNs face is the limited lifespan of sensors. The main causes for this are the battery depletion or the wearing out of the device due to extensive usage. The sensor nodes have the responsibility to emit sensor data and to route sensor data packets until they reach their destination in the network. The sensors consume energy both when sending their own data as well as when forwarding data (working as relay nodes). Sensors which communicate directly with the base station are considered sink nodes as they consequently aggregate data from different sensors. The data collected by the sensors can be hashed, compressed, and then sent directly to the sink node or to a different sensor depending on its location in the network. The study presented in [20] proposes a routing algorithm in the SDWSN context which establishes the packet forwarding routes depending on the energy levels of the nodes in the network. In the traditional WSN, sensors emit data at certain time intervals and in the remaining time, the network stays idle and no processing occurs at the lower sensor level, as presented in [21]. One drawback of using a routing algorithm in an SDN controller environment is that it increases network traffic with control information. First, all the routing decision information has to be aggregated in the controller, and second, the new routing tables must be forwarded to all network devices. As an improvement approach to the previous study, the current work attempts to reduce the communication that is being generated from the sensor level by employing caching techniques, thus maintaining the information locally and performing a few simple constant time procedures on the sensor data. Several caching techniques were experimented in WSNs [16,22]; however, the current work extends the studies by combining caching with content awareness in an SDN controlled environment.

Caching techniques are widely used in the communications domain in order to improve access time to information and to increase network performance. Caching is also a topic of research in the IoT field and more specifically, in the WSN architecture. Content caching has emerged in the mobile environment, most commonly configuring cache locations on a central component (intermediate servers, middleboxes, routers, or gateways) so that the user demands can be accommodated easily without duplicate transmission or redundant communication. The current work extends this approach by proactively delivering the data from the central component towards the application plane and improving the QoS and the QoE of the proposed system. [16] is a study which explores content caching to reduce the energy consumption at certain designated nodes; however, the current work differs in that the content caching takes place at every sensor node. Another enhancement provided by the current research is that it proposes the use of a single type of sensor in the sensors’ plane, therefore simplifying the system design and its deployment.

Traditional routing decisions are performed on information provided by packet headers (source IP address, destination IP address, Time-to-live, etc.). Some studies enhance the routing operations by using different load balancing, scheduling, or clustering techniques [23,24]. The study presented in [25] manages to improve the energy efficiency of an SDWSN by a factor of 20%-40% through a traffic scheduling algorithm. The current work aims to reach a similar performance in reducing traffic volume. [26], again in the context of SDWSN, presents a flow splitting algorithm to minimize traffic load and, therefore, increasing the energy efficiency. Content-aware networking also aims to enhance the routing capabilities by also considering the packet data, not only the header’s information. Content awareness becomes a problem when the information in the payload is encrypted for security reasons, however not all networks are encrypted. Particularly, in the context of WSNs, the resources are of low computational capabilities thus there are situations where no encryption is performed or where it is weak. [27] shows a compromise solution which passes data through simple hashing functions. Passing the same data through a hashing function does not always return the same result as the data is padded differently. However, solutions exist that can determine the similarity of data by processing the hashes [28]. In the context of the current work it is not important if the sensors hash the data or not, as content awareness is implemented only at the sensor node level, and, depending on the content, the sensors will decide to send their data or not. Therefore, the approach proposed by the current research becomes applicable in a multitude of environments, with or without encryption being used.

One of the most used techniques to reduce energy consumption in WSNs is clustering [11,29]. Clustering can be simply described as a method of grouping sensors so that a sensor node, which assumes the responsibility of being a cluster head, will be able to collect data from multiple nearby sensors and send it upstream towards a base station. Clustering techniques choose the cluster head based on the current energy levels of the sensor nodes. Different methods can be employed to determine the cluster heads based on prediction, probability of energy levels [22,30]. A disadvantage of using clustering techniques is that it requires continuous aggregation of energy data from the sensors in order to determine the cluster head, leading to increased network traffic and increased energy consumption. A notable approach is presented in [31] which uses machine learning to establish the sensor clusters for packet forwarding. In contrast, the current work demonstrates the energy efficiency improvements of the proposed methods in a system without clustering techniques deployed. The main goal of the current work is to prove that data transmission can be effectively moved from the data plane to the control plane, thus improving the QoS and QoE for end-user applications.

The measure of success for the proposed approach is given by the improvements in QoS and QoE. QoS can be defined by the improved performance of the system [4], whereas the QoE can be defined as the degree of satisfaction the system manages to provide to end-users [32]. For the proposed approach, a relevant metric to be used is the number of packets which are not transmitted through the WSN layer and are instead generated by the control plane. The network lifetime metric [18], i.e., the time it takes for the first sensor in the network to run out of energy, will be considered. In addition, sensors have different levels of importance: for example, the failure of a sink node has a higher impact in the WSN compared to a normal node, because sink nodes serve as base stations/gateways for all the other nodes in the network. Taking into consideration the above metrics, three main measurements will be presented which demonstrate the impact of the proposed optimizations. The simulations had different network lifetimes, meaning a different number of total transmitted packets for each test. In addition, the importance of sensors placement on sink nodes versus placement on normal nodes is also discussed.

## 3. System Proposal

**Figure 3:**
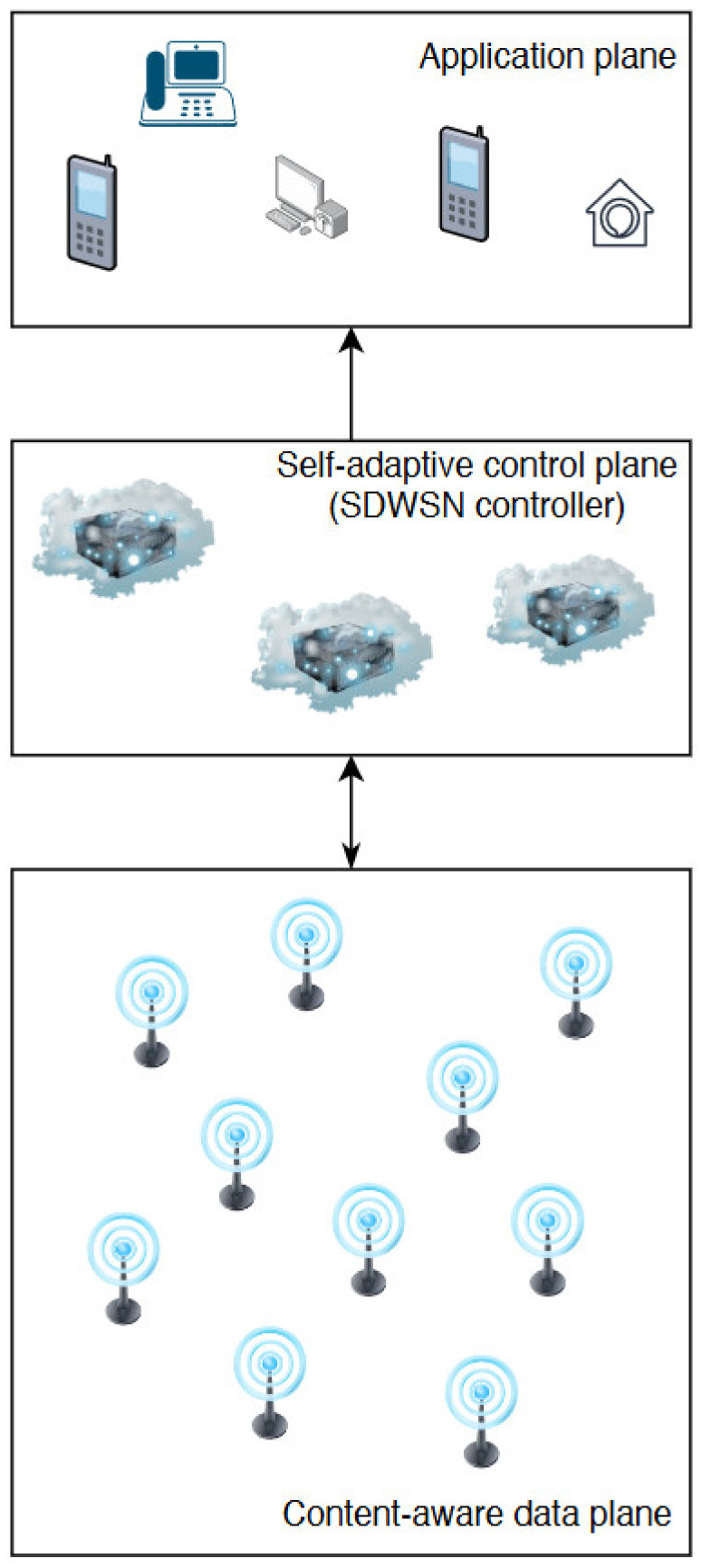
Proposed system architecture.

As previously mentioned, the objective of the current research is to implement a system where a sufficiently knowledgeable control component can manage a WSN and determine whether the sensors should transmit data or not. Additionally, the controller can also decide whether to transmit the data proactively for each sensor, thus reducing the data traffic volume in the WSN layer. This system proposal is presented in Figure 3 For the controller to achieve the proposed functions, it implements two components. First, it requires a basic learning component which learns the behavior for each sensor, i.e., the data transmission frequency for each sensor to determine a pattern. And secondly, it implements a broadcasting mechanism based on the time interval patterns established before. It is important to note that the control component does not collect energy levels from the sensors in order to improve the energy efficiency, it relies solely on the frequency of data transmission from each sensor.

The system can be regarded as being distributed (as the logic is distributed on all the components) and intelligent as it contains a pattern detection module. The communication on the controller’s southbound interface is marked as bidirectional as the controller receives sensor data, but it also coordinates the sensors. On the northbound connection, the traffic is directed only towards the applications. The application plane considers everything beneath it as a service provider, already distributing the data in an acceptable format for the applications.

**Figure 4:**
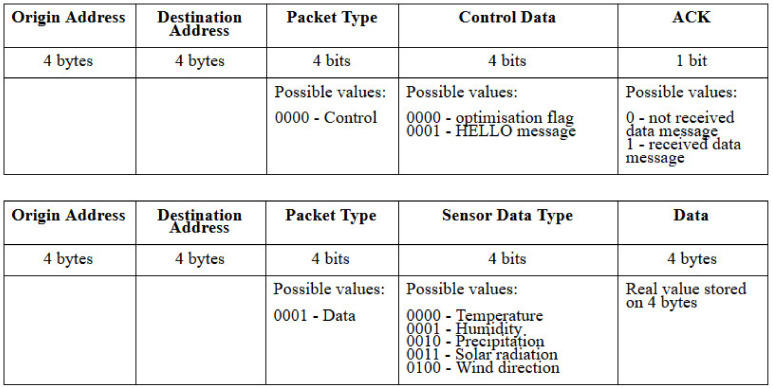
Custom data payload structures for control and sensor data packets.

For the system to work correctly, custom data payloads for control and data packets are employed to allow all the information to be correctly interpreted on all network devices. The custom data payloads are described in Figure 4. However, the proposed solution is general and can be extended for other SDWSN architectures.

The fields and their functions are:

- **Origin Address**: The network device emitting the packet. This can be either sensor data or control messages generated by any network device.
- **Destination Address**: The target destination for the packet. In the case of control data, the destination can be any device in the network. In the case of sensor data, this will always be the controller’s address.
- **Packet Type**: Can be control or sensor data.
- **Control data**: This is only used if the packet type is control data. The current system uses two types of control messages: optimization flag or HELLO message.
- **ACK**: This is only used if the packet type is control data. This field is used to confirm whether the network device has previously received a data packet. This field helps ensure data transmissions without packet loss.
- **Sensor Data Type**: This is only used if the packet type is data. In this case, the field represents the specific type of sensor data (temperature, humidity, precipitation, solar radiation, wind direction), otherwise this field is equal to 0. More details related to the sensor data is given in the Simulations and Results section.
- **Data**: This is only used if the packet type is sensor data. It represents the actual data being delivered (generated by sensors or energy level). The considered sensor data is of real type (single precision). Depending on the hardware architecture the real data type can be stored on 4 or 8 bytes. In embedded resource-constrained environments where the data values are also small, storing this data type on 4 bytes is considered enough, as is also assumed in the current work.

In addition to the above information, the sensor nodes are also configured to transmit HELLO messages [18] periodically to announce their being alive. These messages are only passed between adjacent devices and in case a device stops receiving a few consecutive HELLO messages at the expected time interval it will notify the controller that the sensor which was supposed to send the HELLO message is not reachable anymore. It is at this moment that the network has encountered a problem and the simulation is stopped. In case the controller is the device noticing that its adjacent nodes are not functional, it will directly stop the simulation and end the network lifetime. The HELLO packet’s sending frequency is bigger than the sending of the actual sensor data. This gives the devices enough time to detect whether the adjacent devices are alive or dead so that the controller does not proactively send any data if the sensors have experienced any problems making it unable to send new data.

Following this system overview, the rest of the chapter will focus on the implementation details of the system across the different types of network devices. The system consists of a set of distributed procedures which are either specific to the controller or the sensors or can be common to both types of network devices. The common functionality for both types of network devices is represented by the neighboring mechanism using HELLO messages. The controller operations focus on coordinating the network and proactively deliver the cached data towards the application plane. And finally, the sensor procedures focus on the operation to deliver the sensor information upstream.

Procedure 1 below describes the algorithm for the HELLO messages handling behavior which is implemented at a lower layer on every type of network device (in this case only two, the controller and the sensor types). As the procedure performs a sequence of IF statements which are compiled to a few jump instructions in machine code regardless of the HELLO packet, the complexity of the algorithm is constant time, O(1).
**Procedure 1**. Procedure for HELLO message handling deployed on each network device.**int** helloMisses = 0 **WHILE** (waiting for HELLO packet from network device) **DO**  **IF** (HELLO interval expired **AND** not received packet) **THEN**   ++helloMisses  **IF** (helloMisses > 3) **THEN**   **IF** (current device is controller) **THEN**    **STOP SIMULATION**   **ELSE IF** (current device is sensor) **THEN**    send_device_not_functional_notification   **END**  **END**  **IF** (current device is controller **AND** received device not functional notification)** THEN**   
**STOP SIMULATION**  **END** **END**

Next, the procedures implemented specifically by the control component and the sensor nodes will be described in more detail and exemplified through pseudocode. Also, some implications over the general system behavior are also provided in the explanations.

### 3.1. Control Component Procedures

For the control component to implement the proposed optimizations, it requires the following two procedures: a basic learning component and an adaptive broadcasting component (presented in Procedures 2 and 3).

The operations for the learning component (presented in Procedure 2) defines the use of two map components for storing useful information, one is a map containing the array of each packet received timestamps from each sensor node and the other is a map storing the last received value from each sensor node. When a packet is available for processing, the control component verifies whether it already received data from the specific sensor. If yes, then it will append the packet timestamp to the already existing array of timestamps and if not, it will create the corresponding key for the sensor node in the timestamps map. After inserting the timestamp value in the map, the controller determines whether the packet received timestamps are happening at a constant time interval. If so, the controller will notify the sensor with an optimization flag which will be interpreted by the sensor as being allowed to not send consecutive identical data. Note that the optimization flag is only sent if the sensor has not yet been marked for optimization and the optimization flag is never undone. The complexity of the procedure is logarithmic, O(log n), there are some lookups and insertions performed on the maps containing timestamps and cached data.
**Procedure 2**. Control component algorithm for learning the sensor nodes behavior.**MAP<**sensor, arrayOfIncomingPacketTimestamps>timestampsMap **MAP<**sensor, lastReceivedPacket> cacheMap **WHILE** (check for incoming packet from sensor) **DO**  **IF** (packet is available) **THEN**   **IF** (timestampsMap.CONTAINS(sensor)) **THEN**    timestampsMap.VALUE_OF(packet.node).APPEND(packet.timestamp)   **ELSE**    timestampsMap.ADD(packet.node, packet.timestamp)   **END**   **IF** (sensor not optimized **AND** sensor has constant frequency) **THEN**    send_optimisation_flag_to(packet.origin)   **END**   cacheMap.INSERT(packet.node, packet.data)   **END** **END**

The send_optimisation_flag_to function is implemented as specified in Procedure 3 It determines whether the time intervals are over 1% different than the average values and it requires at least 20 received packets to begin performing calculations, 20 is considered an acceptable data set size to validate the time intervals and regular periodic data transmission intervals are considered. Irregular intervals can be encountered in case of packet transmission errors and in this situation, the learning algorithm will consequently be affected causing delays in notifying marking the sensor nodes for optimization. The more such data transmission errors and delays occur, the longer it will take the learning component to learn the sensor behavior. The complexity of the procedure is considered quadratic, O(n^2^), as the entire array of time intervals between packets is fully iterated for each sensor.
**Procedure 3**. Algorithm to send optimization flag to each sensor. **IF** (COUNT(packets) < 20) **THEN**  **EXIT FUNCTION** 
**END** ATI = SUM(timeIntervalsBetweenPackets) / COUNT(intervals) // ATI = Avg. time Interval **FOR** timeInterval **IN** timeIntervalsBetweenPackets **DO**  **IF** (timeInterval differs from ATI by more than 1%) **THEN**   // do nothing  **ELSE**   mark_sensor_for_optimisation  **END** **END**

After having implemented the above components, all that remains for the Controller is to perform the adaptive data broadcast towards the application plane. Procedure 4 describes the algorithm which starts a timer with the ATI (average timestamp interval) value for each sensor when a packet is delivered to the application plane. Next, the algorithm waits for the time to elapse and in case no new packet is received from the sensor nodes, it will push the cached data (from the cache map populated in Procedure 1) towards the application plane. No proactive push occurs if the control component received any new sensor data, this means that the sensor has generated new different information. The complexity of the algorithm is linear, O(n), as the operations are performed for all of the sensors.
**Procedure 4.** Adaptive data broadcast algorithm **MAP**<sensor, lastReceivedPacket> cacheMap **IF** (packet delivered to applications plane) **THEN**  start_ATI_timer_for_each_optimised_sensor **END** **FOR** sensor **IN** allSensors **DO**  **IF** (sensorIsAlive **AND** sensorMarkedForOptimisation) **THEN**   **WAIT**
**FOR** (reached ATI interval since last emission) **THEN**    **IF** (received new packet from sensor) **THEN**     deliver_new_packet_from_sensor    **ELSE**     broadcast_data_upstream_for_current_sensor(cacheMap, sensor)     **END**    restart_ATI_timer   **END**  **END** **END**

### 3.2. Sensor Nodes Procedures

For the system to implement the proposed optimization, the sensor component must be enhanced with a small amount of logic. This logic is described in Procedures 5 and 6.

Procedure 5 describes the basic algorithm for the sensor transmitting data. When data is available for sending, it first checks whether optimization is enabled or not. In case the optimization is enabled, then the data is only emitted if its value differs from the previously sent value. When data is being delivered by the sensor, the value is cached in the sensor memory for later verifications. In case the values are equal while the optimization flag is set, nothing happens (i.e., the sensor does not transmit the sensor value in order to conserve energy). The data is always emitted in case the optimization has not been enabled by the controller. An additional thing to mention here is the fact that the expected message can be a HELLO message (with or without the ACK bit set). In case the sensor has previously delivered a data packet it will expect that the next received HELLO packet will contain the ACK bit set to 1. This signifies that the data was received correctly on the next sensor. In case the ACK bit is set to 0, then the current sensor will retransmit the data until it received confirmation of successful delivery. As the procedure is only using data from a single network device and it only performs a sequence of IF statements, the complexity of the algorithm is constant time, O(1).
**Procedure 5.** Procedure for sending data.lastEmittedSensorValue = **NULL** waitingForAck = false **WHILE** (check for new packet) **DO**  **IF** (waitingForAck **AND** HELLO packet has ACK == 1) **THEN**   waitingForAck = false;  **ELSE**   resend_previous_data_to_controller  **END**  **IF** (data is available for transmission) **THEN**
   **IF** (optimize data transmission) **THEN**    **IF** (currentPacket != previousPacket) **THEN**     send_data_to_controller      lastEmittedSensorValue = currentSensorData     waitingForAck = true    **END**   **ELSE**    send_data_to_controller    waitingForAck = true   **END**  **END** **END**

Procedure 6 describes the sensor behavior when it receives data from another network device. There are two situations which can be encountered. First, if the received data is the optimization notification from the Controller, the local optimization flag is enabled locally. And second, if the received packet is sensor data generated by another sensor, the sensors perform two operations, first it routes the packet towards the controller and then, it prepares the acknowledge of information receipt to be delivered with the next HELLO packet to the sender. This mechanism, together with Procedure 5, manages to create an environment with no information loss. In case the acknowledgment packets suggest that the packets were lost, they are retransmitted. The procedure complexity can be evaluated to constant time, O(1), as no iterations are performed on any container.
**Procedure 6.** Procedure for receiving data.optimizeDataTransmission = false **WHILE** (check for incoming packet) **DO**  **SWITCH** (packet.type)   **CASE** optimisation_flag:    optimizeDataTransmission = true   **CASE** sensor_data_from_another_sensor:    route_towards_controller    store_packet_for_retransmission_if_needed    prepare_ack_for_sender_in_next_hello_packet  **END**

### 3.3. Packet Exchanges Exemplifications

This subchapter presents the flow of operations in a visual representation in the situations where packet loss does not occur and when it occurs. For simplicity a path containing a controller and 3 nodes is considered for the demonstration.

**Figure 5:**
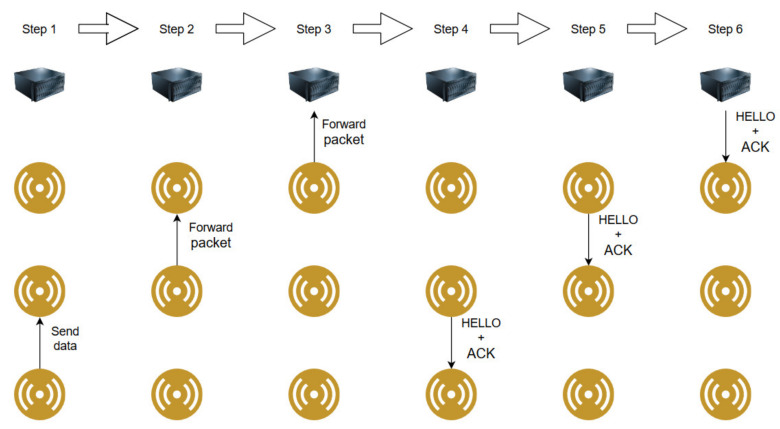
Flow of operations when no packet loss occurs.

First, Figure 5 shows the flow of operations when the data transmission does not present packet loss. The packet is routed to the Controller and the acknowledgments are delivered at a later time together with the HELLO messages. This way, the sensor nodes are instructed to not deliver the data again.

**Figure 6:**
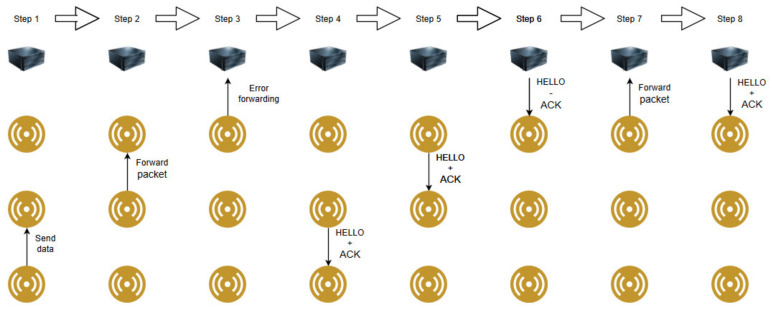
Flow of operations in the resend case when packet loss occurs.

Secondly, Figure 6 shows the flow of operations when the data transmission presents packet loss. The packet is routed to the controller but encounters a transmission error between the last sensor node and the controller (this is visible in Step 3 in the figure). Consequently, in Step 6, the controller is not able to respond with an ACK and therefore, in Step 7 the node attempts to redeliver the sensor data packet. 

One important thing to remark here is what happens in case the controller is broadcasting data upstream during the packet loss. The controller will proactively deliver the previously cached data packet upstream and next, when the resent packet is received, the controller will deliver this packet again. This is not considered to be a problem because in case of delayed transmission it is impossible for the applications plane to receive the data packet any time sooner.

Another clarification is needed about what would happen in the worst-case scenario where transmission errors occur many times consecutively. This problem is solved by implementing a small local buffer (working as a queue) on each node where the payloads are stored as they arrive until they are successfully delivered upstream. This queue is not processing the second element until the first available one is successfully delivered and consequently removed from the queue. Using such a mechanism will ensure that no packets are lost, but it will require that the sensors use a little more memory for correctly implementing the algorithm. However, this would be a problem only in case something goes very wrong in the network, having the HELLO messages being transmitted at a higher frequency than data packets and attempting the retransmission multiple times between data transmissions, the local buffer should normally have a single element to process, therefore not being a big memory consumer.

### 3.4. System Hardware Resources Requirements

Having described the procedures implemented on the devices it is visible that there are some hardware resources requirements which are imposed by the system. Advantageously, these imposed hardware resources are limited to only memory to support the caching feature which is encountered on both the controller and each sensor. Regarding the use of HELLO packets, these are not included in the current proposal’s mathematical model based on the assumption that the use of HELLO messages is already a used mechanism for verifying whether wireless nodes are alive or not. It is guaranteed by the wireless standard and the corresponding literature that during the contention-free period more information can be combined in a single packet, such as data and acknowledgments [33]. However, the extension of these HELLO messages with the ACK bits is mentioned in the model. The network mathematical model is given by the formulas described below. First, we have the data payload size which, according to Figure 4, has different lengths depending on whether the packet contains control or sensor data.

DataPayloadSize = 13 bytes,(1)

(according to Figure 4), next, we must define the network packet size, which is the size of the IP header + the data payload. The minimum size of a TCP packet is 20 bytes [34] and adding it with the data payload size results can have a different value depending on whether the payload represents sensor data or control data. It is also important to note here that the information regarding packet structure may not be valid in heterogenous networks where there are multiple means of devices interconnectivity (not only Wi-Fi).

NetworkPacketSize = TCP_packet_size + data_payload_size,(2)

The energy consumption on the sensor is given by adding the amount of energy it consumes to send a packet, the energy it consumes to receive a packet and the energy it consumes during its idle state:

EnergyConsumptionOnSensor = E(send_packet) + E(receive_packet) + E(idle_state),(3)

In addition to the energy consumption information of the sensor, it also requires an amount of available memory to allow the implementation of the caching mechanism (when comparing the two values for current and previous sensor data values) and to store the necessary routing table for packet delivery both upstream and downstream.

SensorRequiredMemory = 2 * sensor_data_size + payload_queue_size + routing_table_size,(4)

Having described the packets and sensor-related details, the controller is the next component necessary for completely implementing the optimization. It requires more available memory than the sensors as it must cache the last received payload value from every sensor node. The controller also requires a routing table in order to know how to reach all the sensor nodes in the network with control messages (flag for optimization). The formula for determining the controller memory requirements is the following:

ControllerRequiredMemory = nr_sensors * data_payload_size + routing_table_size,(5)

The amount of saved bandwidth in the WSN layer can also be computed with the information of how many packets were saved from transmission multiplied by the network packet size, resulting in the formula:

SavedBandwidthInWSNLayer = COUNT(optimized_packets) * packet_size,(6)

Lastly, the total number of packets flowing through the network is computed by adding the number of all generated packets (from every sensor) and the number of total forwarded packets (again from every sensor):

TotalNumberOfPackets = COUNT(generated_packets) + COUNT(forwarded_packets) + COUNT(resent_packets) + COUNT(ACK_bits_delivered),(7)

## 4. Testing Environment

**Figure 7:**
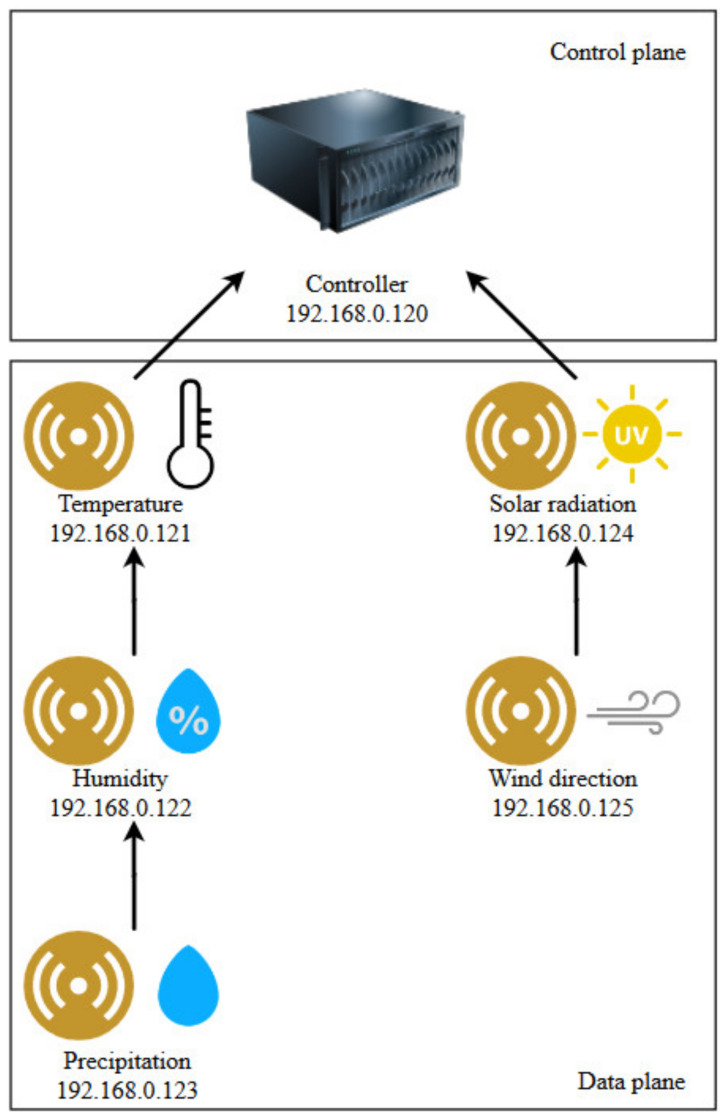
Test network topology, adapted from [18].

The test environment presented in Figure 7 was constructed by implementing a control component and a generic sensor component. The control component and several sensor instances were deployed to different virtual machines on different computers in a wireless local area network (WLAN). It is also possible for initial testing to configure multiple network devices in the same machine by using virtual network interfaces, however, a wireless hop between the network devices is considered mandatory in this use case to bring the environment closer to a WSN.

Each sensor implements features to generate sensor data and to act as a router according to the illustrated connections between network devices. As opposed to using simulators such as Mininet [35], where all the network devices are run from a single computer, this approach has the advantage that there is a more realistic time interval between the packet emission and arrival. In addition, each sensor can be easily configured to generate random data or to generate already stored data from an online source at any interval.

The network is IP enabled and static IP addresses were set on the network devices according to the network topology. The sensors were implemented with modules to generate sensor data, generate HELLO packets and routing capabilities to forward packets to a destination. Sensor data packets on the network devices were generated from real sensor data retrieved from data sources [36] over a span of four weeks. The HELLO packets were designed to be generated at a much higher frequency than the sensor data packets.

The routing table is statically configured on each network device in order to simplify the implementation and, the purpose of this research is not routing, but data transmission optimization. Sensors were configured to send data at approximately regular intervals; however, each sensor was initialized on startup with a random interval for data transmission so that the learning component on the controller uses a dynamic mechanism for learning the frequency patterns.

The components were implemented in C++ with the help of the Qt framework [37] for the facilities it brings to network communication and software writing in general. The reason for choosing C++ under Qt is that Qt is a cross-platform framework allowing the code to be compiled and run with ease on any type of operating system. In addition, C++ code can be easily compiled into a library which would be used in any future research using simulators such as OMNeT++ [38] or Mininet [35] under any environment. An additional reason for running simulations under C++ is that this language is arguably one of the most performant in terms of computation speed/data processing especially for resource-constrained environments, such as SDWSNs.

## 5. Simulations and Results

The simulated context was the climate difference in different geographical regions. To be noted here that the system works in other contexts as well, weather was chosen as the test topic because there are many data sources readily available which can be used to perform tests. Historical weather data was taken from the data source website referenced in [36] for different locations: Reykjavik (Iceland), Ouarzazate (Morocco), Budapest (Hungary). The reason for choosing these three locations is due to the different climates that they present. Reykjavik has an oceanic rainy climate, Ouarzazate has a desertic climate, whereas Budapest has a temperate continental climate. Having different data patterns, different results were obtained by employing the system following a discussion which is the best environment to deploy this system in. Each sensor was attributed one weather parameter, having five sensors, the five weather attributes that were considered were: temperature, humidity, precipitation, solar radiation, wind direction. The total sensor data spans four weeks (4 May–31 May 2020) and collected sensor data happened at one-hour intervals.

Next, several individual simulation scenarios are presented where networks with different lifetimes were created in the before mentioned locations and the impact of the implemented optimizations is calculated in each scenario. To simulate the different network lifetimes, the sensors were configured to stop sending HELLO messages at different moments in time. An in-depth analysis is performed between the two scenarios (non-optimized and optimized) which were performed on the same data set. In addition, several simulations were also performed with different packet loss ratios to illustrate the performance of the proposed solution in less ideal situations.

### 5.1. Long Network Lifetime

**Figure 8:**
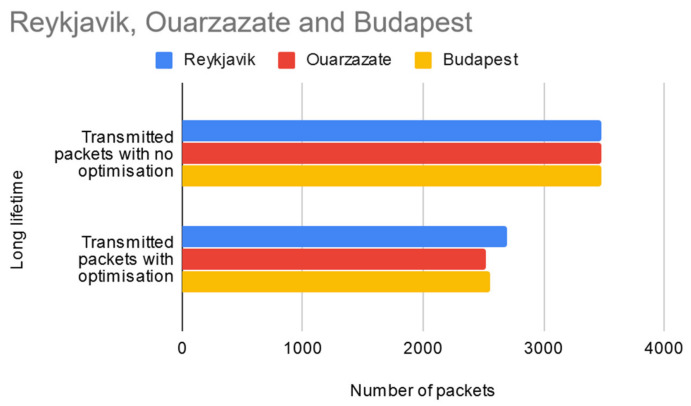
Test results for long network lifetime scenario.

The first simulation scenario (presented in Figure 8) supposes the longest network lifetime in the current tests. In this case, the HELLO messages are always transmitted, therefore the network doesn’t die in this case, the sensor data sample is fully used. From a total of 3480 packets which would normally have been delivered by the sensor nodes for each of the three regions, the implemented optimization manages to reduce this number to 2697 for Reykjavik, 2522 to Ouarzazate and 2549 for Budapest, therefore saving a total of 2672 packets in the WSN layer. This translates into 2672 × 33 = 88,176 bytes of data (according to Equations (2) and (6) above) not transmitted in the WSN layer, therefore increasing the sensors’ lifespan by reducing their energy consumption when generating sensor data. An additional benefit is the fact that less generated packets also means that nodes closer to the controller must perform fewer forwarding operations thus prolonging their lifetime.

### 5.2. Medium Network Lifetime

**Figure 9:**
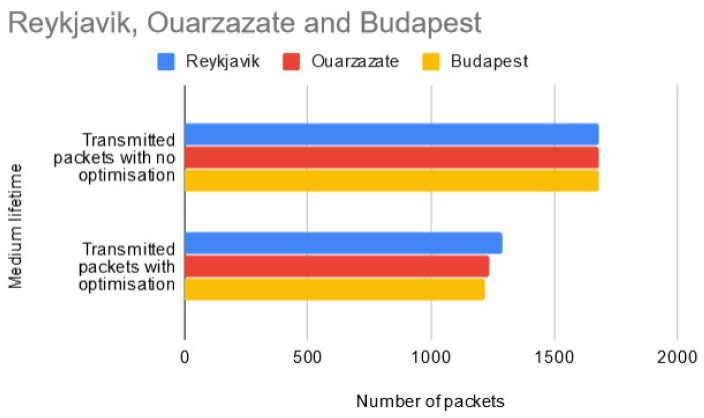
Test results for medium network lifetime scenario.

In the second test scenario (presented in Figure 9), the network lifetime was configured to end when the entire data sample size was half depleted. This is the moment when the sensor nodes were configured to stop sending the HELLO packets. From a total of 1680 packets which would normally have been delivered by the sensor nodes for each of the three regions, the implemented optimization manages to reduce this number to 1288 for Reykjavik, 1237 to Ouarzazate and 1219 for Budapest, therefore saving a total of 1296 packets in the WSN layer. This translates into 1296 × 33 = 42,768 bytes of data (according to Equations (2) and (6) above) not transmitted in the WSN layer.

### 5.3. Short Network Lifetime

**Figure 10:**
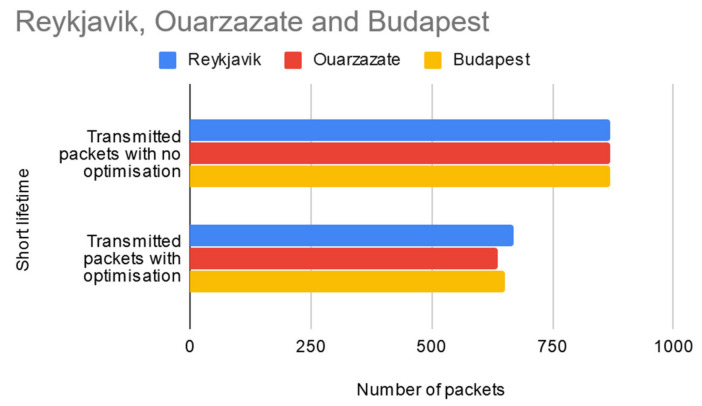
Test results for short network lifetime scenario.

In the third test scenario (presented in Figure 10), the lifetime of the network was reduced to about a quarter of the size of the initial data set until the sensors were configured to stop sending HELLO packets. From a total of 870 packets which would normally have been delivered by the sensor nodes for each of the three regions, the implemented optimization manages to reduce this number to 669 for Reykjavik, 637 to Ouarzazate, and 650 for Budapest, therefore saving a total of 654 packets in the WSN layer. This translates into 654 × 33 = 21,582 bytes of data (according to Equations (2) and (6) above) not transmitted in the WSN layer.

### 5.4. Detailed Sensor Traffic Analysis

**Figure 11:**
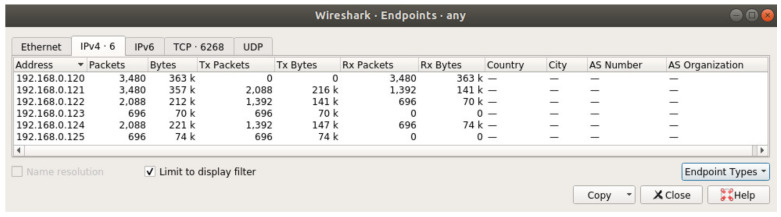
Statistics for non-optimized simulation.

**Figure 12:**
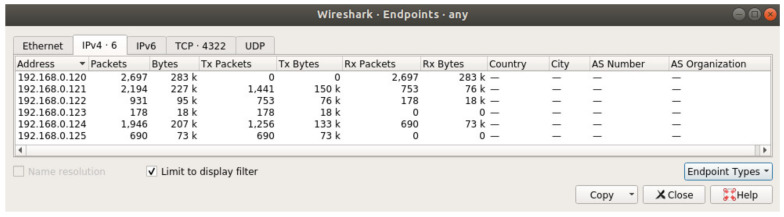
Statistics for optimized simulation.

This subchapter presents a more detailed analysis of sensor data traffic. Figure 11 and Figure 12 show sensor traffic statistics captured using Wireshark when simulations were run on a single computer using multiple virtual network interfaces (as opposed to running the system on multiple network devices in the other scenarios). The complete data set for Reykjavik was run twice in the system, the first time without optimization and the second time with optimization. The reason these two simulations were run on a single computer was to allow the generation of Wireshark displayable statistics. The results are not altered in any way as the system behaves the same way in both simulation environments.

In Figure 11, which shows the results for a non-optimized simulation, it can be seen on the Tx Packets column (transmitted packets column) that the leaf sensors (identified by the IP address corresponding with Figure 7) transmit 696 packets. Their parents are transmitting 1392 which is a doubled 696 value. The reason for this is that the parents of the leaf nodes act as forwarding nodes as well as data generation nodes, so they are generating 696 packets, but they also forward the packets generated by the leaf nodes. Consequently, advancing towards the root node of the network, each sensor collects more packets to forward upstream.

Figure 12 shows the results for an optimized simulation of the same data set as used in Figure 8. The transmitted packets column (Tx Packets) shows fewer packets, but the behavior that the number of packets is increasing as approaching the root node is still present. An important issue to point out, which becomes more visible in this capture, is the fact that the more packets the leaf nodes are generating the more forwarding traffic will exist in the network. Therefore, another optimization which could be made is to rearrange the network so that the sensors which are emitting fewer packets are placed as close as possible to the leaf level of the network. Ideally, using Equation (3) above the energy level (given by the number of packets) should be as low as possible and using Equation (7) the total number of packets should be as low as possible in the network.

### 5.5. Results Analysis and Interpretation

**Figure 13:**
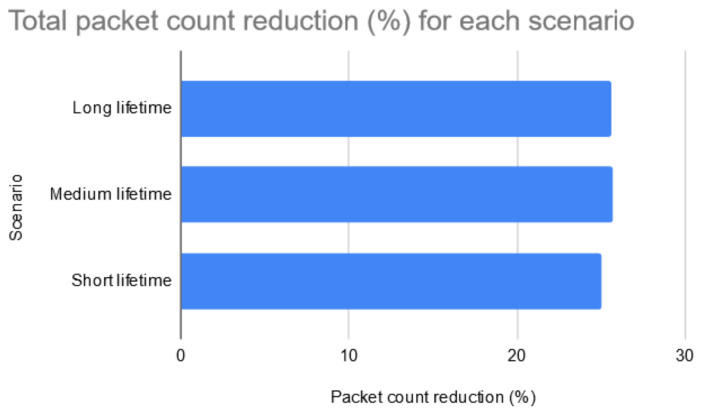
Gain in reducing the number of generated packets with no transmission errors.

The computation of the total gains (presented in Figure 13) for the different network lifetime scenarios reveal that the proposed system presents a consistent behavior across each scenario. In the long lifetime scenario, the total gain (i.e., the percentage of optimized packets) represents 25.1% of all packets. In the medium lifetime scenario, the total gain represents 25.71% of the total number of packets. And finally, in the short lifetime scenario, the gain represents 25.05% of all packets. This leads to the conclusion that the system saves approximately 25% of the generated network traffic. Most of the saved data values which are not delivered are the default sensor values which are generated when the sensors have nothing to report and they confirm this by sending empty values. The slight difference across each scenario depends solely on the nature of the sensor data in the selected data set.

Although overall the results all have an approximately identical gain percentage, looking at location results across different lifetime scenarios there are some differences. For example, in the long lifetime scenario, Ouarzazate has a better performance than Budapest, but the roles are reversed in the medium lifetime scenario. In addition, by inspecting detailed analysis with Wireshark it can be deduced that this system would be more suitable for implementation in certain natural environments more than others. For example, the temperature values are more constant in Reykjavik than in Ouarzazate due to the climate, however, the precipitation situation is reversed between the two locations. In this case, the system could be deployed only for certain types of sensors/ or the sensors could be arranged differently in the network tree as mentioned in the previous subchapter. Additional benefits could be generated when working with integer data, instead of real data. At the moment, values such as 10.1 and 10.15 are considered different but depending on the system requirements where this implementation is deployed and the needed accuracy, sensor values might already be rounded and, therefore, they might generate an even greater performance gain. Nevertheless, even if some environments are more suitable than others, the system proves to bring benefits in any environment it is implemented in.

### 5.6. Scenarios with Packet Loss

The previous scenarios were run considering there is no packet loss or packet retransmission. Having presented them, it would also be interesting to analyze the behavior in case packet loss does occur. Since there are multiple types of packets that can be lost (packet that notifies optimization, data packet, acknowledgement packet) they can increase the network traffic with different proportions. In case a data packet or the optimization packet are lost, the network device will simply retransmit the packet because the acknowledgment was not received. This will increment the number of delivered packets by one (because retransmission only occurs from the previous host). However, if an acknowledgment packet is lost this will cause the transmitting device to resend the packet again towards the destination, thus duplicating the entire traffic on the path, having a bigger impact on the network compared to the previous situation. This second scenario can also affect the calculation of the learning component which is based on the frequency of arrived packets potentially delaying the decision to mark a sensor for optimization due to irregular data flows.

**Figure 14:**
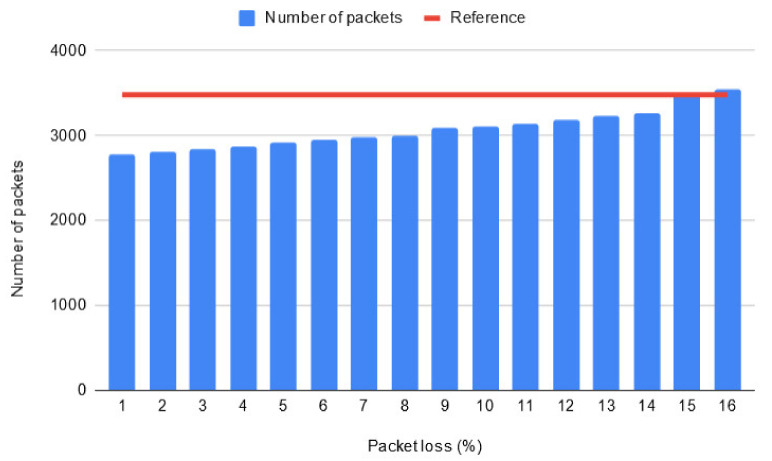
Statistics for the scenarios with packet loss.

Next, simulation results are presented for multiple test runs where packet loss was configured to occur with a random chance in increments of 1%. The purpose of this test is to find out what error rate makes the proposed optimization less efficient than the scenario where no optimization has occurred considering that the literature describes a percentage of 5% to be critical for the QoS of a system [39]. Figure 14 displays statistics obtained after running the simulations for Reykjavik (long network lifetime) with different percentages of packet loss.

The simulations were run 10 times for each percentage value and the average obtained total number of packets is shown in the graph. Due to the nature of chance, the more runs which can be performed, the more consistent and reliable the findings will be. It can be observed that, on average, the proposed solution stops behaving more efficiently when the packet loss transmission rate reaches 15%. At this point, the average total number of transmitted packets is 3492, which is greater than the reference point 3480 (which was previously obtained when no optimization was configured in the system). However, it is important to mention that a test run for the scenario with a 14% chance of packet loss resulted in 3512 total packets (which is higher than the reference value), but this was an isolated case as the average number of total packets was 3257 in this case. Another important thing to note is that as the chance of error increases, the results become more volatile. For example, in the test runs with a 1% error chance, the lowest number of total packets was 2767 and the highest number was 2815, resulting in a difference of 48 between the two values. Whereas in the test runs with a 15% error chance, the lowest number of total packets was 3288 and the highest number was 3807, resulting in a difference of 491 between the two values.

## 6. Discussions and Conclusions

Enabling a WSN with an SDN component brings many opportunities for network optimizations. The current work focused on reducing the energy consumption of a WSN by removing duplicated traffic and moving it in the control plane of the SDWSN. The system proves to be effective in removing the duplicated traffic in the WSN layer through a few simple procedures implemented on both the controller and the sensor nodes by combining concepts such as learning (on the controller), content awareness (on the sensor nodes) and caching. The system is efficient even in environments which present packet loss demonstrated by the previous test runs where transmission errors occur. The threshold where the system becomes less efficient is when the packet transmission rate reaches 15%, which is a lot more than the threshold defined in the literature for experiencing poor QoS in wireless data systems. The additional needed memory resources for the system are negligible in the WSN layer. The controller does require more memory to cache data from each sensor in the topology; however, the controller does not belong to a resource constrained environment and it can be more easily upgraded to support the implementation. In addition to the reduction of the energy consumption in the WSN layer, this system proposal presents an additional benefit, namely reducing the congestion on the sensor node buffers due to reduced network traffic, thus speeding up the packet processing.

In the test scenarios, employing data from three real-life locations it can be argued that the system is more appropriate for implementation in a location more than it would be for other locations due to the actual data values which can be more stable in some parts of the globe. However, this is by no means a rule in every possible scenario, as the test data can be different depending on what period is selected. In any case, one definitive conclusion that can be drawn is that the system brings optimizations in any environment it is implemented in. It also is successful in increasing the network lifetime, as fewer packets get transmitted by the WSN layer, it is improving the QoS by reducing traffic load in the WSN layer and improves the QoE by proactively delivering cached data towards the applications layer instead of waiting for the same data to be received from the sensors. There are some limitations and situations where QoE is not achieved, but it is detailed in the next paragraph.

Due to the nature of the proposed system where the acknowledgments for data transmission arrive at the sending device only together with the next HELLO packet, the system presents some delays in confirmation of packet arrival and data retransmission in case of packet loss. This can be considered a limitation of the system design and consequently it may not be suitable for time-critical environments (such as military or defense). Nevertheless, the system does ensure that no packets are lost during the network lifetime and is reliable in gathering sensor data in any non-time-critical environments. Another problematic case which can be considered a limitation of the system is the delayed receipt of data in the applications plane when packet loss occurs along the way. In addition, during the retransmission attempts, the controller can reach the timeout to proactively forward the cached data upstream and when the delayed transmission finally arrives on the controller, the controller will deliver the new data as well. What could be considered a problem is the fact that the controller delivers the cached data again right before receiving the new actual data, thus increasing the network load. However, this operation does not take place in a resource-constrained environment anymore and it should not have a big impact on the network operations. The QoE can be negatively impacted here in the fact that the data delivered to the applications plane is not updated to the latest value, but this would behave the same way with or without the proposed system. In all other cases, the QoE is positively affected as the data values are delivered faster to the end-users.

As for future work, the control component could be extended with a more complex machine learning component taking into consideration not only the data transmission frequency but also parameters such as payload content, congestion levels in various places of the WSN, detailed sensors energy levels, etc. The network traffic can also be optimized by employing header compression to also reduce the packet size, not just the number of packets being transmitted. In addition, the system can be implemented with more dynamic data emission patterns. Another option would be to also extend the algorithm with an adaptive routing protocol. The current implementation presents a realistic WSN behavior as between every neighboring network device a wireless hop was present during the tests, however, it would be insightful to see this system implemented with real sensors.
